# Predictors of dropout in the German disease management program for type 2 diabetes

**DOI:** 10.1186/1472-6963-12-8

**Published:** 2012-01-10

**Authors:** Birgit Fullerton, Antje Erler, Boris Pöhlmann, Ferdinand M Gerlach

**Affiliations:** 1 Institute of General Practice, Johann Wolfgang Goethe-University, Theodor-Stern-Kai 7, 60590 Frankfurt, Germany; 2 AQUA Institute for Applied Quality Improvement and Research in Health Care GmbH, Maschmühlenweg 8-10, 37073 Göttingen, Germany

## Abstract

**Background:**

To improve and assess the effectiveness of disease management programs (DMPs), it is critical to understand how many people drop out of disease management programs and why.

**Methods:**

We used routine data provided by a statutory health insurance fund from the regions North Rhine, North Wurttemberg and Hesse. As part of the German DMP for type 2 diabetes, the insurance fund received regular documentation of all members participating in the program. We followed 10,989 patients who enrolled in the DMP between July 2004 and December 2005 until the end of 2007 to study how many patients dropped out of the program. Dropout was defined based on the discontinuation of program documentation on a particular patient, excluding situations in which the patient died or left the insurance fund. Predictors of dropout, assessed at the time of program enrolment, were explored using logistic regression analysis.

**Results:**

5.5% of the patients dropped out of the disease management program within the observation period. Predictors of dropout at the time of enrolment were: region; retirement status; the number of secondary diseases; presence of a disabling secondary disease; doctor's recommendations to stop smoking or to seek nutritional counselling; and the completion and outcome of the routine foot and eye exams. Different trends of dropout were observed among retired and employed patients: retired patients of old age, who possibly drop out of the program due to other health care priorities and employed people of younger age who have not yet developed many secondary diseases, but were recommended to change their lifestyle.

**Conclusions:**

Overall, dropout rates for the German disease management programs for type 2 diabetes were low compared to other studies. Factors assessed at the time of program enrolment were predictive of later dropout and should be further studied to provide information for future program improvements.

## Background

In Germany, disease management programs for type 2 diabetes (DMP DM 2) were introduced on a nationwide scale in 2003. All German statutory health insurance (SHI) funds, which cover about 85% of the German population [1,2], are required, by law, to offer these programs, though participation is voluntary for doctors and patients. Over the years, DMP participation has been increasing continuously. To date, there are about 3.4 million patients enrolled in the DMP DM 2 in Germany, which are approximately 40% to 60% of all diabetic patients in Germany [3,4]. Key components of German DMPs are evidence-based treatment guidelines, patient training, the regular documentation of disease development and treatment goals, as well as provider feedback. Usually, general practitioners (GPs) act as coordinating physicians: they enrol patients into the program, organize patient training, and negotiate individual treatment goals with patients [5,6].

The evaluation of German DMPs is a legal requirement: it is carried out according to a pre-determined design based on data from routine documentation forms that doctors have to submit at regular intervals for every enrolled patient [7,8]. This official evaluation program has been criticized for its methods, such as the lack of a control group, which makes it difficult to assess effectiveness of the programs [9,10].

Recently, several studies showed improvements in care, such as adherence to treatment guidelines and the completion of regular routine exams, as well as patient and clinical outcomes such as quality of life and mortality [5,11-14] (but also see [15]).

However, in many of the studies potential biases are insufficiently addressed. One aspect that has been ignored in the official evaluation program, as well as in some of the recent studies, is the rate of attrition [5,8,14]. Understanding the mechanisms of patients' dropout from DMPs is important to evaluate potential bias due to attrition in studies that look at the effectiveness of DMPs [16,17]. Furthermore, improved knowledge of how many and which kind of patients are likely to drop out of the DMP is important to decide whether and how the design of these programs could be improved to avoid losing patients who could potentially benefit from disease management.

Therefore, in this study we wanted to specifically focus on dropout from DMPs. We aimed to investigate, firstly, how many people who initially enrolled in a DMP left the program again at a later time. Secondly, to better understand the underlying reasons, we looked at which variables assessed at the time of enrolment were predictive of later dropout.

## Methods

### Data

To investigate the research question we used anonymized routine DMP data from the Techniker Krankenkasse (TK), a large German SHI fund, from the regions Hesse, North Rhine, and North Wurttemberg from the years 2004 to 2007. Doctors who participate in a DMP are required to fill in a documentation form for every enrolled patient every three or six months depending on the severity of the disease. There are separate forms for enrolment of a patient and follow-up documentation. The official evaluation institute receives the full documentation data, which includes some clinical data, such as blood pressure, HbA1c, and medication. SHI funds, however, only receive a reduced dataset. Our analysis is based on this reduced dataset, which includes information on: diabetes duration; whether the patient has diabetes-specific symptoms; use of diabetes medication; the diagnosis of secondary diseases (hypertension; stroke; lipid disorder; coronary hear disease (CHD); nephropathy; retinopathy; neuropathy; peripheral artery disease (PAD); blindness; myocardial infarction; amputation; diabetic foot; dialysis); the foot status; the number of hypoglycaemic events; the number of hospital admissions due to hyperglycaemia within the last twelve months; whether the patients has participated in or was recommended to take part in training; whether the patient has received an annual ophthalmological exam; whether the patient was given recommendations to stop smoking or seek nutritional advice; and therapy goals such as to alter or hold the HbA1c or blood pressure.

Data from the DMP documentation were linked with the routine claims data from the insurance fund to obtain further information on patient age, gender, region, insurance status and whether the patient had died or left the insurance fund during the observation period.

### Study design

We used a study design of a retrospective cohort study. Our study population consisted of all patients who enrolled in the DMP for the first time between July 1^st ^2004 and December 31^st ^2005. We did not include any enrolments before July 2004, because different documentation forms were used. Patients who died or left the insurance fund before the end of 2007 were excluded from the analysis. Patients were followed-up until the end of 2007. The outcome of interest was whether patients would drop out of the DMP. Dropout was defined as a patient having no DMP documentation (no follow-up documentation but also no new enrolment) for at least all of the last year of follow-up (2007). For the remainder of the article, patients with missing DMP documentation in 2007 are referred to as the dropout group; the other patients are referred to as the DMP group.

### Data analysis

All baseline data at the time of DMP enrolment was analyzed descriptively by calculating absolute and relative frequencies for categorical variables and means and standard deviations for continuous variables. The chance of dropout was modelled using logistic regression. For the regression models, secondary diseases were summarized in the variables 'two or more secondary diseases' and 'disabling secondary disease', whereby stroke, blindness, amputation and dialysis were considered disabling secondary diseases. All variables were first analyzed using univariate models. Additionally a multivariate logistic model was built using backward elimination and forward selection. Finally, we also added the variables age and gender to the final multivariate model independent of whether they showed any association with the outcome. Model performance was evaluated using the Hosmer-Lemeshow test. Patterns of health care utilization have been observed to be different for younger employed and for retired elderly patients: Retired elderly patients highly value a close and continuous relationship to their GP [18]), while younger, employed patients have shown to be more critical towards their primary health care provider and less adherent to his recommendations [19]. Therefore, we also carried out the analysis stratified by insurance status.

Patients with missing values in any of the variables were excluded from the analysis. The frequencies of missing values in all variables were compared between the two groups of patients.

## Results

**Figure 1:**
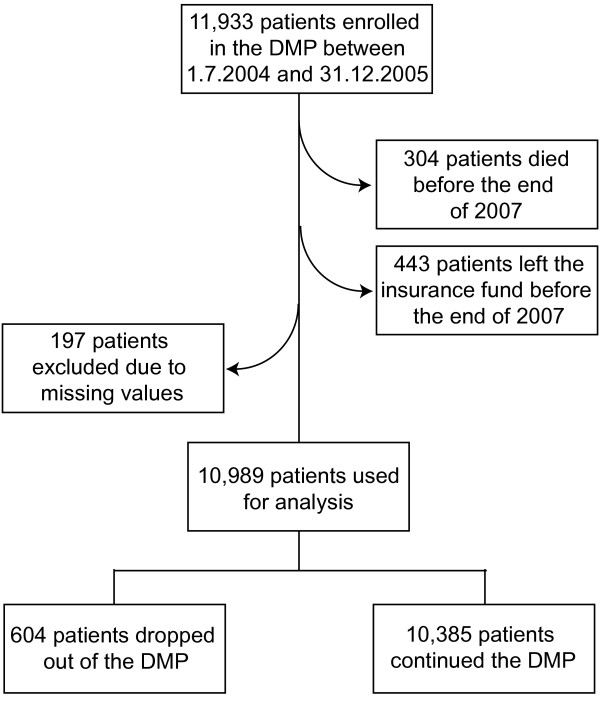
**Flow chart of data processing**.

Overall, 11,933 patients enrolled in the DMP Diabetes mellitus type 2 between 1.7.2004 and 31.12.2005. 304 patients died and 443 patients left the insurance fund before the end of 2007 - they were excluded from the analysis. Of the remaining 11,186 patients a further 197 cases were excluded due to missing data (see figure 1). From the final group of 10,989 patients, 5.5% (604) dropped out of the DMP within the observation period.

### Missing data

For 197 patients we found missing values in six of the baseline variables (see table 1). Overall, the amount of missing data was low and the distribution of missing values was similar in the dropout group compared to the DMP group. Therefore, we accepted the assumption that values were missing completely at random as plausible and only performed a complete case analysis.

**Table 1:** Frequency of missing values across variables

	DMP (n = 10571)	Dropout (n = 615)	total (n = 11186)
Insurance status	109 (1.0%)	5 (0.8%)	114 (1.0%)
Blood pressure treatment goal	1 (0.0%)	0 (0.0%)	1 (0.0%)
Foot status	1 (0.0%)	0 (0.0%)	1 (0.0%)
Diabetes duration	74 (0.7%)	6 (1.0%)	80 (0.7%)
Hospital admissions due to hyperglycaemia	2 (0.0%)	0 (0.0%)	2 (0.0%)
Gender	109 (1.0%)	5 (0.8%)	114 (1.0%)

### Baseline

The baseline data from the time of DMP enrolment are shown in table 2. The mean age of the study population was 63.2 (SD = 10.0) years and 67.5% were male. This high representation of men in our study population is likely due to the fact that the TK traditionally was a SHI fund for people with technical professions. On average, patients had lived with diabetes for 6.1 years and the majority was taking some form of diabetes medication (68.5%). 17.6% had not yet developed any secondary diseases, but 19.5% suffered from more than one secondary disease. 20.3% of the patients had already participated in a diabetes training program before enrolment in the DMP; only 0.2% had participated in a hypertension training program. 35.2% of the patients were recommended by their doctor to seek nutritional counselling and 11.1% were recommended to stop smoking. For 49.6% of the patients, the treatment goals included lowering the HbA1c and 30.8% of the patients aimed to lower their blood pressure. The annual eye exam - for which the DMP guidelines demand that the patient should be referred to a specialist - had been completed in 39.1% of the cases and was already scheduled in another 37.1%.

**Table 2:** Baseline data

	DMP (n = 10385)	Dropout (n = 604)	Overall (n = 10989)
Age - Mean (SD)	63.3 (9.8)	62.6 (12.5)	63.2 (10.0)
Diabetes duration in years - Mean (SD)	6.1 (6.7)	6.0 (6.7)	6.1 (6.7)
Gender - n (%) male	6998 (67.4)	414 (68.5)	7412 (67.5)
Region - n (%)			
Hesse	3042 (29.3)	169 (28.0)	3211 (29.2)
North Wurttemberg	1175 (11.3)	92 (15.2)	1267 (11.5)
North Rhine	6168 (59.4)	343 (56.8)	6511 (59.3)
Insurance status - n (%)			
member	3815 (36.7)	261 (43.2)	4076 (37.1)
retired	5960 (57.4)	310 (51.3)	6270 (57.1)
family	610 (5.9)	33 (5.5)	643 (5.9)
Hypertension - n (%)	7147 (68.8)	391 (64.7)	7538 (68.6)
Stroke - n(%)	427 (4.1)	7.6 (5.8)	462 (4.2)
Lipid disorder - (%)	4377 (42.2)	37.6 (37.6)	4604 (41.9)
CHD - n (%)	1578 (15.2)	84 (13.9)	1662 (15.1)
Nephropathy - n (%)	468 (4.5)	22 (3.6)	490 (4.5)
Retinopathy - n (%)	393 (3.8)	28 (4.6)	421 (3.8)
Neuropathy - n (%)	986 (9.5)	65 (10.8)	1051 (9.6)
PAD - n (%)	575 (5.5)	33 (5.5)	608 (5.5)
Blindness - n (%)	17 (0.2)	4 (0.7)	21 (0.2)
Myocardial infarction - n (%)	661 (6.4)	32 (5.3)	693 (6.3)
Amputation - n (%)	41 (0.4)	4 (0.7)	45 (0.4)
Diabetic foot - n (%)	128 (1.2)	12 (2.0)	140 (1.3)
Dialysis - n (%)	20 (0.2)	1 (0.2)	21 (0.2)
No secondary diseases - n (%)	1817 (17.5)	112 (18.5)	1929 (17.6)
Two or more secondary diseases - n (%)	2034 (19.6)	105 (17.4)	2139 (19.5)
Disabling secondary disease^1 ^- n (%)	487 (4.7)	41 (6.8)	528 (4.8)
Diabetes symptoms - n (%)	3019 (29.1)	193 (32.0)	3212 (29.2)
Diabetes medication - n (%)	7115 (68.5)	410 (67.9)	7525 (68.5)
Severe hypoglycaemic episodes within the last 12 months - n (%)	121 (1.2)	12 (2.0)	133 (1.2)
Hospital admissions due to hyperglycaemia - n (%)	66 (0.6)	5 (0.8)	71 (0.7)
Foot status - n (%)			
normal	9244 (89.0)	513 (84.9)	9757 (88.8)
abnormal	423 (4.1)	36 (6.0)	459 (4.2)
not assessed	718 (6.9)	55 (9.1)	773 (7.0)
Diabetes training received - n (%)	2129 (20.5)	105 (17.4)	2234 (20.3)
Diabetes training recommended - n (%)	3257 (31.4)	217 (35.9)	3474 (31.6)
Hypertension training received - n (%)	26 (0.3)	0 (0.0)	26 (0.2)
Hypertension training recommended - n	866 (8.3)	45 (7.5)	911 (8.3)
(%)			
Nutritional counseling recommended - n (%)	3628 (34.9)	243 (40.2)	3871 (35.2)
Recommendation to stop tobacco use - n (%)	1128 (10.9)	87 (14.4)	1215 (11.1)
HbA1c treatment goal - n (%)			
hold	5229 (50.4)	286 (47.4)	5515 (50.2)
lower	5136 (49.5)	316 (52.3)	5452 (49.6)
raise	20 (0.2)	2 (0.3)	22 (0.2)
Blood pressure treatment goal - n (%)			
hold	7193 (69.3)	415 (68.7)	7608 (69.2)
lower	3192 (30.7)	189 (31.3)	3381 (30.8)
Annual eye exam - n (%)			
done	4102 (39.5)	199 (33.0)	4301 (39.1)
scheduled	3831 (36.9)	244 (40.4)	4075 (37.1)
not done	2452 (23.6)	161 (26.7)	2613 (23.8)

SD: standard deviation, CHD: coronary heart disease, PAD: peripheral artery disease, All variables refer to the time of programme enrolment. ^1 ^Stroke, amputation, blindness and dialysis were considered disabling secondary diseases.

### Logistic regression models

The results of the univariate logistic regression models are shown in table 3. These variables were then used for building a multivariate logistic model. Two methods of variable selection, forward selection and backward elimination, resulted in the same model, which included the variables region, insurance status, number of secondary diseases, presence of a disabling secondary disease, recommendation to stop smoking, recommendation to seek nutritional counselling, performance of the annual eye exam, and examination of the foot status. The multivariate odds ratios shown in table 4 are based on a logistic model that in addition to these variables also includes the variables age and gender, even though these variables did not show a strong association with dropout. The results suggest that the probability of dropout from the DMP is lower for patients from North Rhine or Hesse compared to patients from North Wurttemberg. Patients who were already retired at the time of enrolment into the DMP were also less likely to leave the DMP compared to full insurance members, who are usually employed. The presence of several secondary diseases at the time of enrolment was generally associated with a decreased probability of dropout, unless these secondary diseases were of a disabling nature, in which case the probability of dropout was increased. Dropout was also associated with treatment goals set at the time of enrolment. Patients who were recommended to stop smoking or to seek nutritional counseling were at an increased risk of dropout. Whether the DMP guidelines-recommended regular exams were carried out also seemed to influence the probability of later dropout. People who had already visited an ophthalmologist for the recommended yearly eye exam were less likely to leave the DMP compared to patients for whom the exam was not even planned. It is also part of the DMP guidelines that doctors examine the feet of diabetic patients. If the exam had not been carried out at enrolment or if the foot status was abnormal compared to a normal result, we found the probability of dropout to be increased.

**Table 3:** Univariate logistic models of dropout

	OR (95%-CI)
Age	
10 years difference	0.94 (0.86, 1.02)
Diabetes duration in years	
5 years difference	0.99 (0.93, 1.05)
Gender	
male	1
female	0.95 (0.80, 1.13)
Region	
North Wurttemberg	1
North Rhine	0.71 (0.56, 0.90)
Hesse	0.71 (0.55, 0.92)
Insurance status	
member	1
retired	0.76 (0.64, 0.90)
family	0.79 (0.55, 1.15)
2 or more secondary diseases	
< 2	1
≥ 2	0.86 (0.70, 1.07)
Disabling secondary disease^1^	
no	1
yes	1.48 (1.06, 2.06)
Diabetes symptoms	
no	1
yes	1.15 (0.96, 1.37)
Diabetes medication	
no	1
yes	0.97 (0.82, 1.16)
Severe hypoglycaemic episodes within the last 12 months	
no	1
yes	1.72 (0.95, 3.13)
Hospital admissions due to hyperglycaemia	
no	1
yes	1.31 (0.52, 3.25)
Foot status	
normal	1
abnormal	1.53 (1.08, 2.18)
not assessed	1.38 (1.04, 1.84)
Diabetes training received	
no	1
yes	0.82 (0.66, 1.01)
Diabetes training recommended	
no	1
yes	1.23 (1.03, 1.46)
Hypertension training recommended	
no	1
yes	0.89 (0.65, 1.21)
Nutritional counseling recommended	
no	1
yes	1.25 (1.06, 1.48)
Recommendation to stop tobacco use	
no	1
yes	1.38 (1.09, 1.75)
HbA1c treatment goal	
hold	1
lower	1.13 (0.95, 1.33)
raise	1.83 (0.43, 7.86)
Blood pressure treatment goal - n (%)	
hold	1
lower	1.03 (0.86, 1.23)
Annual eye exam - n (%)	
done	1
not done	1.35 (1.09, 1.68)
scheduled	1.31 (1.08, 1.59)

All variables refer to the time of programme enrolment. ^1 ^Stroke, amputation, blindness and dialysis were considered disabling secondary diseases.

**Table 4:** Multivariate logistic model of dropout

	OR (95% CI)	p-value
Age		
10 years difference	1.05 (0.95; 1.18)	0.3476
Gender		
male	1	
female	1.01 (0.83;1.22)	0.9607
Region		
North Wurttemberg	1	
North Rhine	0.70 (0.55; 0.89)	0.0032
Hesse	0.71 (0.54; 0.92)	0.0099
Insurance status		
member	1	
retired	0.74 (0.59; 0.92)	0.0075
family	0.80 (0.53; 1.18)	0.2575
Number of secondary diseases		
< 2	1	
≥ 2	0.77 (0.61; 0.98)	0.0351
Disabling secondary disease^1^		
no	1	
yes	1.79 (1.26; 2.56)	0.0013
Recommendation to stop smoking		
no	1	
yes	1.32 (1.03; 1.68)	0.0262
Recommendation to seek nutritional counseling		
no	1	
yes	1.22 (1.03; 1.45)	0.0222
Ophthalmological exam		
done	1	
not done	1.32 (1.06; 1.64)	0.0131
scheduled	1.26 (1.04; 1.53)	0.0197
Foot status		
normal	1	
abnormal	1.66 (1.16; 2.37)	0.0059
not assessed	1.35 (1.01; 1.81)	0.0437

All variables refer to the time of programme enrolment. ^1 ^Stroke, amputation, blindness and dialysis were considered disabling secondary diseases.

Table 5 shows the odds ratios of the same logistic regression model as described above, but stratified according to insurance status. Since the number of people with insurance status 'family member' is low, we only show the results for members and retired persons. While age showed no significant association with dropout in the overall model, it shows opposite effects in the two different membership subgroups: for retired patients higher age is associated with a higher probability of dropout, while for people who are still employed, older people are less likely to drop out of the DMP. Regarding the presence of secondary diseases, both groups show similar trends. While the recommendation to stop smoking seems to have more effect within the 'member' group, it seems that the effects regarding routine eye and foot examinations mainly apply to the group of retired patients.

**Table 5:** Predictors of dropout stratified by insurance status

	Employed OR (95% CI)	Retired OR (95% CI)
Age		
10 years difference	0.82 (0.70; 0.95)	1.55 (1.30; 1.84)
Gender		
male	1	
female	0.91 (0.65;1.28)	1.04 (0.82; 1.32)
Region		
North Wurttemberg	1	
North Rhine	0.70 (0.48; 1.00)	0.69 (0.50; 0.97)
Hesse	0.57 (0.38; 0.86)	0.80 (0.55; 1.15)
Number of secondary diseases		
< 2	1	
≥ 2	0.67 (0.43; 1.05)	0.87 (0.65; 1.16)
Disabling secondary disease^1^		
no	1	
yes	1.88 (0.90; 3.92)	1.64 (1.08; 2.49)
Recommendation to stop smoking		
no	1	
yes	1.44 (1.05; 1.96)	1.21 (0.79; 1.86)
Recommendation to seek nutritional counseling	1	
no	1.28 (0.99; 1.66)	1.25 (0.99; 1.60)
yes		
Ophthalmological exam		
done	1	
not done	1.19 (0.84; 1.70)	1.46 (1.09; 1.95)
scheduled	1.39 (1.03; 1.89)	1.13 (0.86; 1.48)
Foot status		
normal	1	
abnormal	1.60 (0.81; 3.14)	1.75 (1.14; 2.68)
not assessed	1.03 (0.63; 1.68)	1.58 (1.08; 2.32)

All variables refer to the time of programme enrolment. ^1 ^Stroke, amputation, blindness and dialysis were considered disabling secondary diseases

## Discussion

In this study we identified a number of factors measured at the time of patient enrolment into the DMP DM 2 that were associated with later dropout from the program. We found that patients from North Wurttemberg tended to dropout from the DMP more frequently compared to patients from other regions. This result might reflect the different levels of acceptance of DMPs in different regions in Germany [20]. In the region North Rhine for example, similarly structured programs for the management of diabetes had existed long before the current DMPs were introduced [21]. Here, the acceptance of disease management programs was high and implementation quick. Most patients who had participated in the structured diabetes programs were then enrolled in the DMP DM 2. On the contrary, in North Wurttemberg, resistance against the introduction of DMPs from doctors was high, which might have also affected continuity in patients' DMP participation. Dropout was further associated with insurance status, number of secondary diseases, presence of a disabling secondary disease, treatment goals requiring behavioral/lifestyle changes (such as the recommendation to stop smoking or seek nutritional counselling) and the completion of regular exams required by the DMP guidelines. Based on an additional analysis stratified according to insurance status (see table 5), we could observe different trends for the group of patients who were still working and those who were retired. Among the employed, those who were younger, healthier and recommended by their doctor to change their lifestyle were especially at risk of later dropping out of the DMP. Among the retired however, predictors of dropout were older age, the presence of a disabling secondary disease, an abnormal foot status or routine exams not being carried out. These could possibly reflect two very different reasons for dropout. First, for younger people who are still fairly healthy and pre-occupied with their working life, DMPs might not be perceived as beneficial, but rather the requests to make changes to their lifestyle might be perceived as an inconvenience. Second, retired people of older age might also be at higher risk of dropout, since other health-related issues might obtain higher priority; patients might be close to the end of their life, spend more time in hospital or move to long-term care. The observation that not carrying out the required routine exam(s) was also associated with dropout in this group could possibly also be a reflection of the doctor's decision that due to other circumstances in the life of the patient, these exams might not be necessary anymore. Further research would be needed to explore these trends of dropout in more detail.

Overall, we found the percentage of patients dropping out of the DMP DM 2 to be low (5.5%) in our study. However, we only followed patients over a short time period and our ability to measure dropout accurately might have been limited. Dropout rates in international studies on disease management vary widely. In a recent meta-analysis including 41 randomized controlled trials on disease management interventions with varying durations from 1.5 to 48 months, dropout rates ranged between 1.1 and 39.8% (mean = 14.0%) [22]. The results from this meta-analysis refer to dropouts of clinical trials, which might not reflect real-world conditions and might therefore not be comparable to dropout from disease management programs outside of study conditions. However, similarly varied results can be found in studies looking at attrition rates from diabetes education services, either at physician-led diabetic clinics or diabetes education centres usually lead by diabetes educators. In studies carried out in Britain, the United States, Canada, Ireland, and Japan, dropout rates ranged from 4 to 57% per year [23,24]. Due to the diversity of programs as well as variations in assessing attrition, comparison between studies is difficult. Enrolment in the German DMPs is voluntary. Furthermore, inclusion criteria for the German DMPs are the willingness and ability of the patient to actively participate in the programme and an expected benefit for the patient in terms of an improvement in quality of life and life expectation. The GP as coordinating physician decides about a patient's suitability for the programme. Therefore DMP participants are likely to be a selected group in that they might be more motivated and able to reach their treatment goals, take responsibility to self-manage their chronic disease, and are adherent to treatment recommendations. Patients with low motivation or ability to follow recommendations to improve the management of their disease would be enrolled in the DMP less frequently because of a lack of potential benefit. This could explain why the overall rate of drop-out was low in our study.

Regarding predictors of dropout, our results are consistent with the results from international studies. As in our primary analysis, most studies did not find a relationship between attrition and gender, age or diabetes duration [24]. If we stratified for retirement status, however, we found an association between attrition and age. As in our results, retirement status itself was significantly related to dropout in other studies [24,25]. Also, in qualitative studies, in which patients were asked why they discontinued program participation, conflicts with work was a reason frequently given [23]. Other reasons given were low perceived concern for the disease, not considering attendance important, not feeling sick, but also having too many other health conditions [23,25]. In our data we found a similar trend, as patients suffering from a disabling secondary disease as well as patients with fewer secondary diseases were more likely to dropout of the program. The association of dropout with recommendations to stop smoking or seek nutritional counselling has not been explicitly investigated in other studies; however, results on smoking status and dietary treatment in relation to the likelihood of dropout were contradictory.

Other variables, which have been shown to be related to patient dropout and were not addressed in our study, were socio-economic status, patients' perceptions, attitudes and beliefs as well as variables related to the program provider, such as physician-specific variables, practice structure and organization [23-25].

### The definition of dropout

Since it is not part of the DMP routine documentation to record when a patient ends his or her participation in a DMP, and since we only relied on routine data in this study without asking patients or doctors directly, we used a definition of dropout based on the presence or absence of submitted DMP documentation forms. As described above, there are two types of documentation forms, one for enrolment in the DMP and one for follow-up. We found that 40.7% of the patients had one or more documentation of enrolment after their initial entry into the DMP. In this study we ignored these re-enrolments, as we do not know whether the use of an additional enrolment form necessarily implies that the patient had left the DMP and then enrolled again. There also might be other reasons for why a doctor submits a new enrolment documentation instead of a follow-up documentation: for example, in the DMP DM 2, until July 2008, a new enrolment form needed to be submitted every time a patient changed to a new doctor, in which case a new enrolment form might have been used even though the patient never interrupted his or her participation in the DMP. Another situation in which a second enrolment would occur is if a patient missed two subsequent follow-up documentation intervals, or if the patient without plausible reason did not participate in patient training within 12 months after it had been recommended to him or her twice. In this situation the patient would be removed from the DMP by the health insurance, but could re-enrol at a later time. We investigated the patterns of re-enrolments in more detail and found that in 31.1% (1,561) of the cases, re-enrolment was associated with a change to a new doctor, and in 30.8% (1,547) the patient was missing at least two documentation intervals before the time of re-enrolment, indicating removal from the DMP and later re-enrolment. In 9.1% (457) we observed both a change of doctor and a documentation break of two or more intervals preceding re-enrolment. However, for 47.2% (2,370) of all re-enrolments, we could not identify any possible reason for why these patients should have been re-enrolled - they neither changed doctor nor did they miss enough appointments to be excluded from the DMP (see table 6). For this reason we decided not to differentiate between enrolment and follow-up documentation forms in this study and defined dropout solely based on the absence of any kind of submitted DMP documentation in 2007.

**Table 6:** Re-enrolments and missed appointments

Possible reasons for re-enrolment	% (n)
Changed to new doctor	31.1 (1,561)
Missed previous 2 documentation intervals	30.8 (1,547)
Changed to new doctor and missed previous 2 documentation intervals	9.1 (457)
No reason identified	47.2 (2,370)

**Missed documentation intervals**	

At least one missing documentation	60.6 (6,662)
≥ 20% of documentations missing	29.5 (3,240)

We also did not take into account to what extent patients actually participated in the program as intended. There may have been some patients who, although officially enrolled, missed most of their appointments (since in our definition of dropout, the number of missed appointments did not matter). In theory, a patient could have enrolled in the DMP once in 2004 or 2005, and then not participate in the DMP until 2007 and would not have been considered a dropout as long as he or she had at least one DMP documentation form in 2007. In our data, we found that in fact 60.6% (6,662) of all patients had at least one missing routine documentation and 29.5% (3,240) had missed 20% or more of the required appointments (see table 6). We would argue that the extent to which patients actually participate in the DMP as planned is a separate issue from that of discontinuing participation completely and likely would have led to different results.

Since patients ending their DMP participation is not documented as part of the routine data collection, we also do not know who initiated a dropout from the DMP: was it the patient's decision to leave the program, was it the doctor's decision, or did program participation end due to external circumstances, such as a long stay in rehabilitative care; in which case the insurance fund would have ended the DMP participation automatically after a period of missing follow-up documentation intervals.

### Strengths and weaknesses of the study

To our knowledge, this study is the first that explicitly examined dropout from DMPs in Germany. We believe that looking at attrition should be an important aspect of the evaluation of DMPs: firstly, because the attrition rate can be a strong indicator of the success of the program, secondly, because understanding which people are likely to dropout and for which reasons can provide valuable information for the improvement of DMPs, and thirdly, because not taking attrition into account appropriately when evaluating the effectiveness of DMPs could lead to biased results.

The main weaknesses of this study are related to the use of routine data. We know only very little about the validity of the data that is collected routinely as part of DMPs. The regional data collection agencies carry out completeness and plausibility checks, but to our knowledge no one has yet carried out external validity checks based on other data sources such as the patients' medical records. Due to the use of routine data, we also have little direct understanding of the reasons for why the patient left the program. Furthermore, we were only able to look at a limited number of variables provided in the routine data set. However, the use of routine data allowed us to study a large number of patients, which would have not been feasible otherwise.

Another limitation of our study is the short duration of the follow-up period: we only studied people who left the DMP a short time after enrolment. A very different group of people might remain in the DMP for a longer period and only decide much later to discontinue their participation. Finally, we only studied data from one sickness fund in three regions in Germany. The level of interest in DMPs has been shown to vary widely regionally and between different sickness funds [20,26,27]. Therefore, our results may not be representative for all of Germany.

## Conclusions

Overall dropout from the German disease management program DM 2 seems to be low, but variables assessed at the time of enrolment are predictive of future dropout. We could identify two trends of dropout: firstly, retired patients of old age, who possibly drop out of the DMP due to other health care priorities towards the end of life and secondly, employed people of younger age who have not yet developed many secondary diseases, but were recommended to change their lifestyle. Both patterns of dropout are important for the evaluation of the effectiveness of DMPs, since they could bias the results if not taken into account appropriately. The second trend should be of particular interest for the design of DMPs, since it applies to a group of patients who could benefit the most from preventive measures offered as part of the DMP. Further research is needed to investigate in more detail to what extent the DMP DM 2 can successfully include patients at earlier stages of disease and if this may help them to take preventive measures to avoid disease progression.

## Competing interests

The authors declare that they have no competing interests.

## Authors' contributions

BF designed the study, analyzed the data and drafted the manuscript. AE reviewed different stages of the manuscript. All authors reviewed and approved the final manuscript.

## Pre-publication history

The pre-publication history for this paper can be accessed here:

http://www.biomedcentral.com/1472-6963/12/8/prepub

## References

[B1] Federal Ministry of Health KM 6 statistics (statutory health insurance: insured persons) 2010 http://www.gbe-bund.de/gbe10/hrecherche.prc_herkunft_rech?tk=51310&tk2=51312&p_fid=9156&p_uid=gastr&p_aid=1858634&p_sprache=E&cnt_ut=1&ut=51312 (Last accessed: 4.4.2011)

[B2] Federal Statistical Office Update of the state of the population Federal Statistical Office 2010 http://www.gbe-bund.de/gbe10/hrecherche.prc_herkunft_rech?tk=51310&tk2=51311&p_fid=402&p_uid=gastr&p_aid=1858634&p_sprache=E&cnt_ut=1&ut=51311 (Last accessed: 4.4.2011)

[B3] International Diabetes Federation IDF Diabetes Atlas 2009 Brussels, Belgium: International Diabetes Federation

[B4] Diabetes DE Deutscher Gesundheitsbericht: Diabetes 2011 2011 Mainz: Kirchheim + Co GmbH

[B5] Stock S Drabik A Buscher G Graf C Ullrich W Gerber A Lauterbach KW Lungen M German diabetes management programs improve quality of care and curb costs Health Aff (Millwood) 2010 29 2197 2205 10.1377/hlthaff.2009.0799 21134920

[B6] Siering U Nolte E, Knai C, McKee M Germany Managing Chronic conditions: experience in eight countries 2008 Copenhagen: World Health Organization on behalf of the European Observatory on Health Systems and Policies 75 96

[B7] Bundesversicherungsamt Kriterien des Bundesversicherungsamtes zur Evaluation strukturierter Behandlungsprogramme 2009 http://www.bundesversicherungsamt.de/cln_108/nn_1046154/DE/DMP/Downloads/Downloads__Evaluation__gesamt.html (Last accessed: 4.4.2011)

[B8] Bundesversicherungsamt Bericht des Bundesversicherungsamtes zur vergleichenden Evaluation von strukturierten Behandlungsprogrammen bei Diabetes mellitus Typ 2 2009 http://www.bundesversicherungsamt.de/cln_115/nn_1046154/DE/DMP/Downloads/Evaluationsergebnisse__DM2__03-06__bericht.html (Last accessed: 4.4.2011)

[B9] Birnbaum D Braun S [Evaluation of Disease Management Programmes - assessing methods and initial outcomes from a health economic perspective] Z Evid Fortbild Qual Gesundhwes 2010 104 85 91 10.1016/j.zefq.2009.07.002 20441013

[B10] Fullerton B Nolte E Erler A [The quality of chronic care in Germany ] Z Evid Fortbild Qual Gesundhwes 2011 105 554 62 10.1016/j.zefq.2010.12.025 22142877

[B11] Miksch A Laux G Ose D Joos S Campbell S Riens B Szecsenyi J Is There a Survival Benefit Within a German Primary Care-Based Disease Management Program? Am J Manag Care 2010 16 49 54 20148605

[B12] Ose D Wensing M Szecsenyi J Joos S Hermann K Miksch A Impact of Primary Care-Based Disease Management on the Health-Related Quality of Life in Patients With Type 2 Diabetes and Comorbidity Diabetes Care 2009 32 1594 10.2337/dc08-2223 19509007 PMC2732141

[B13] Schäfer I Kuever C Gedrose B Hoffmann F Ruß-Thiel B Brose HP van den Bussche H Kaduszkiewicz H The disease management program for type 2 diabetes in Germany enhances process quality of diabetes care-a follow-up survey of patient's experiences BMC Health Services Research 2010 10 55 10.1186/1472-6963-10-55 20199685 PMC2843701

[B14] Ullrich W Marschall U Graf C Versorgungsmerkmale des Diabetes Mellitus in Disease Management Programmen Diabetes, Stoffwechsel und Herz 2007 16 407 414

[B15] Linder R Ahrens S Köppel D Heilmann T Verheyen F Nutzen und Effizienz des Disease-Management-Programms Diabetes mellitus Typ 2 Dtsch Ärztebl 2011 108 155 162 21475573 10.3238/arztebl.2011.0155 PMC3071961

[B16] Conklin A Nolte E Disease management evaluation: A comprehensive review of current state of the art 2011 Santa Monica, CA: RAND Corporation 10.7249/rhq-01-01-07 PMC4945214 28083163

[B17] Linden A Adams J Roberts N Evaluation methods in disease management: determining program effectiveness Position Paper for the Disease Management Association of America (DMAA) 2003

[B18] Berkelmans PGJ Berendsen AJ Verhaak PFM Van der Meer K Characteristics of general practice care: What do senior citizens value? A qualitative study BMC Geriatr 2010 10 80 10.1186/1471-2318-10-80 21044316 PMC2984451

[B19] Klingenberg A Bahrs O Szecsenyi J Wie beurteilen Patienten Hausärzte und ihre Praxen. Deutsche Ergebnisse der europäischen Studie zur Bewertung hausärztlicher Versorgung durch Patienten (EUROPEP) Z Arztl Fortbild Qualitatssich 1999 93 437 445 10519193

[B20] Nagel H Baehring T Scherbaum WA Diabetesversorgung: Deutliche regionale Unterschiede Dtsch Ärztebl 2006 103 390 39921910264

[B21] Altenhofen L Haß W Oliveira J Modernes Diabetesmanagement in der ambulanten Versorgung. Ergebnisse der wissenschaftlichen Begleitung der Diabetesvereinbarung in der KV Nordrhein Wissenschaftliche Reihe des Zentralinstituts fur die Kassenarztliche Versorgung in der Bundesrepublik Deutschland 2002 57 Köln: Deutscher Ärzte-Verlag

[B22] Pimouguet C Le Goff M Thiebaut R Dartigues JF Helmer C Effectiveness of disease-management programs for improving diabetes care: a meta-analysis CMAJ 2010 183 E115 E127 21149524 10.1503/cmaj.091786 PMC3033953

[B23] Griffin SJ Lost to follow-up: the problem of defaulters from diabetes clinics Diabet Med 1998 15 Suppl 3 S14 S24 **S14-24** 9829764 10.1002/(sici)1096-9136(1998110)15:3+<s14::aid-dia725>3.3.co;2-9

[B24] Gucciardi E A systematic review of attrition from diabetes education services: strategies to improve attrition and retention research Can J Diabetes 2008 32 53 65

[B25] Gucciardi E Demelo M Offenheim A Stewart DE Factors contributing to attrition behavior in diabetes self-management programs: a mixed method approach BMC Health Serv Res 2008 8 33 10.1186/1472-6963-8-33 18248673 PMC2277391

[B26] Busse R Disease management programs in Germany's statutory health insurance system Health Aff (Millwood) 2004 23 56 67 15160803 10.1377/hlthaff.23.3.56

[B27] Erler A [Introduction of disease management programmes in Germany as reflected by differing interests of health insurance companies and the federal association of statutory health insurance physicians] Gesundheitswesen 2002 64 572 577 10.1055/s-2002-35538 12442215

